# [Expression of Concern] *In vitro* and *in vivo* targeting of bladder carcinoma with metformin in combination with cisplatin

**DOI:** 10.3892/ol.2026.15741

**Published:** 2026-07-02

**Authors:** Dong Wang, Xiaohou Wu

Oncol Lett 10: 975–981, 2015; DOI: 10.3892/ol.2015.3267

Following the publication of the above paper, it has been drawn to the Editor's attention by a concerned reader that, regarding the immunohistoochemical images shown in Fig. 4 on p. 978, two pairs of overlapping data panels were identified in panels B-D, such that the data in these three panels were appearently derived from the same original source, where different experimental conditions were reported.

The authors have been contacted by the Editorial Office to offer an explanation for these apparent anomalies in the presentation of the data in this paper, and we are awaiting their response. Owing to the fact that the Editorial Office has been made aware of potential issues surrounding the scientific integrity of this paper, we are issuing an Expression of Concern to notify readers of these potential problems while the Editorial Office continues to investigate this matter further.

